# Cardiac adverse events associated with lacosamide: a disproportionality analysis of the FAERS database

**DOI:** 10.1038/s41598-024-67209-0

**Published:** 2024-07-13

**Authors:** Chengcheng Yang, Wanqi Zhao, Huihui Chen, Yinhui Yao, Jingmin Zhang

**Affiliations:** 1 Department of Pharmacy, The First People’s Hospital of Shangqiu, Shangqiu, 476000 China; 2 The University of Manchester, Manchester, M13 9PL England; 3 Department of Pharmacy, Chengde Medical University Affiliated Hospital, Chengde, 067000 China; 4 Department of Pharmacy, Henan Key Laboratory for Precision Clinical Pharmacy, The First Affiliated Hospital of Zhengzhou University, Zhengzhou, 450052 China

**Keywords:** Lacosamide, Cardiac adverse events, Disproportionality analyses, FAERS database, Reporting odds ratio, Drug safety, Toxicology

## Abstract

Lacosamide was the first approved third-generation antiepileptic drug. However, real-world data regarding its adverse cardiac reactions in large samples still need to be completed. We evaluated the cardiac safety profile of lacosamide using the Food and Drug Administration Adverse Event Reporting System (FAERS). We performed disproportionality analysis computing reporting odds ratio (ROR) as a quantitative metric to assess the signal of lacosamide-related cardiac adverse events (AEs) from 2013 Q1 to 2022 Q4. The signal was considered significant when the lower limit of the 95% confidence interval (CI) of the ROR exceeded 1, and ≥ 5 AEs were reported. Serious and nonserious cases were compared by statistical analysis, and signals were further prioritized using a rating scale. A total of 812 cardiac AEs associated with lacosamide were identified, and 92 signals were detected, of which 17 AEs were significantly associated signals. The median time-to-onset (TTO) for moderate priority signals was 10 days, whereas for weak priority signals, it was 54 days. Notably, all cardiac AEs exhibited an early failing pattern, indicating the risk gradually decreasing. Based on the comprehensive analysis of the FAERS database and prioritization of cardiac AE signals, our research enhances the awareness among healthcare professionals regarding cardiac AEs associated with lacosamide.

## Introduction

Lacosamide (LCM), as an *N*-methyl-d-aspartate (NMDA) receptor glycine site-binding antagonist, is approved by the European Medicines Agency (EMA) and US Food and Drug Administration (FDA) for the treatment of focal (partial-onset) seizures for adults 17 years and older in 2008, and it was extended down to 4 years of age in 2017^1–3^. LCM is a chiral functionalized amino acid with a unique mechanism of action that can selectively enhance the slow inactivation of voltage-gated sodium channels (VGSC), effectively reducing sodium influx and decreasing neuronal excitability^4^. VGSC is a vital ion channel responsible for action potential generation^5^. Previous clinical trials have shown good tolerability and efficacy of adjuvant treatment with LCM^6^. However, in more than 10 years of use, its adverse reactions have gradually been reported^7^.

Antiepileptic drugs are recognized to cause adverse effects on specific organs and systems. In addition to the nervous system, VGSC are abundant in cardiac tissue and play a crucial role in maintaining normal rhythm. Theoretical considerations suggest that sodium channel blockade, as seen with some antiepileptic drugs, could lead to side effects on the heart, affecting its functioning^8^. For its cardiac adverse effects, sinus bradycardia, atrioventricular block, ventricular tachycardia, atrial fibrillation and atrial flutter have been reported successively after appearing on the market of LCM^8,9^. Several large-scale studies have found that cardiac adverse events (AEs) associated with the oral administration of LCM, with an overall incidence ranging between 0.7% and 1.2%^9,10^. Recent investigations have highlighted an increase in cardiac adverse effects in patients undergoing LCM treatment. Current studies have found that cardiac adverse reactions are weak and mild, and most of them do not require intervention or can be relieved after drug withdrawal^11,12^. However, in the real world, the application of LCM may be accompanied by underlying diseases and face many individual differences. There are also case reports of serious AEs caused by LCM due to cardiac adverse reactions^13,14^. A review of research findings by FDA in 2021 suggested that patients with heart disease who take epilepsy and mental health drug lamotrigine may have an increased risk of arrhythmias. It is desirable to assess whether other drugs in the same class (e.g., carbamazepine, LCM) have similar effects on the heart. Safety studies on these drugs are recommended^15^. Furthermore, FDA labels specify that LCM is contraindicated in patients with pre-existing atrioventricular block second degree and atrioventricular block complete and that it is prone to atrial arrhythmias (atrial fibrillation or flutter), especially in patients with diabetic neuropathy and/or cardiovascular disease^16^.

As one of the largest pharmacovigilance databases in the world, the FDA Adverse Event Reporting System (FAERS) is a public, voluntary, and spontaneous reporting database designed to facilitate the FDA's post-market safety monitoring of drugs and therapeutic biologic product. The present clinical trials are constrained by various limitations, including the representation of specific populations, small sample sizes, restricted follow-up periods, and stringent inclusion and exclusion criteria. The relationship between LCM and cardiac adverse reactions may not reflect real-world circumstances adequately, making the detection of rare and serious cardiac adverse reactions challenging. Therefore, it is beneficial to employ the FAERS monitoring system to investigate the adverse reactions of LCM. While there are alternative databases and methods available for identifying AEs, conducting disproportionation analysis of cardiac adverse reactions using FAERS data represents one viable approach among several for further research in this area. The FAERS database, widely employed to identify real-world pharmacoalert risk signals, was utilized in this study to extract reports of LCM-related cardiac adverse reactions, analyzing cardiac adverse reaction data, AE records, and drug usage records of patients prescribed LCM^17^.In order to understand the real-world of cardiac adverse reactions associated with LCM, and thereby offer guidance for its safe clinical use in treating epilepsy patients, it is essential to conduct further investigations.

## Results

### Descriptive analysis

A total of 14,980,109 AE reports were collected between January 1, 2013 and December 31, 2022. After deduplication, 9,738 AEs related to LCM were reported, of which 812 (8.33%) were cardiac AEs (Table 1). In addition, out of the total AEs related to LCM, there were 1,795 unique reports, among these, 133 unique reports specifically contained cardiac AEs. Moreover, 725 AEs were combined with other drugs. Levetiracetam, valproic Acid and lamotrigine were commonly combined drugs, they were recorded for 243 (29.93%), 76 (9.36%) and 60 (7.39%). The top 20 combined drugs were shown in Table S5. The incidence of cardiac-related AEs was slightly higher in women than in men (52.86% vs 47.14%). The age distribution was dominated by patients over 18 years old (93.41%), among which the number of cases in the 18–64 age group (47.31%) was more, and the median age was 62 years old. The interquartile range (IQR) was 37–75. Most cardiac-related AEs occurred in individuals weighing less than 80 kg (69.69%), with a median weight of 68 kg (54–82 kg). Most reported cases originated from the United States (n = 308, 37.93%), followed by Japan (n = 128, 15.76%). Compared with all AEs caused by LCM, cardiac adverse reactions accounted for a higher proportion of serious AEs (74.29% vs. 91%), with other significant medical events comprising 73.88% (n = 546) of these events, prolonged hospitalization accounting for 43.17% (n = 319), and death outcomes making up 17.46% (n = 129).

**Table 1:** Clinical characteristics of patients with LCM -associated AEs.

Characteristics	LCM induced cardiac AEs (n = 812)		LCM induced overall AEs (n = 9738)	
	Available number	Value	Available number	Value
Gender, n (%)	734 (90.39)		8692 (89.26)	
Female		388 (52.86)		4743 (54.57)
Male		346 (47.14)		3949 (45.43)
Age (years), n (%)	577 (71.06)		5433 (55.79)	
< 18		38 (6.59)		653 (12.02)
18 ≤ and ≤ 64		273 (47.31)		3264 (60.08)
> 64		266 (46.10)		1516 (27.90)
Median (IQR)		62.00 (37.00–75.00)		49.00 (29.00–66.00)
Weight (kg), n (%)	287 (35.34)		2536 (26.04)	
< 80		200 (69.69)		1783 (70.3)
80 ≤ and ≤ 100		65 (22.65)		476 (18.77)
> 100		22 (7.67)		277 (10.92)
Median (IQR)		68 (54–82)		68 (53–84)
Reported countries, n (%)	812 (100)		9738 (100)	
US		308 (37.93)		6202 (63.69)
JP		128 (15.76)		866 (8.89)
DE		77 (9.48)		476 (4.89)
FR		77 (9.48)		424 (4.35)
CO		17 (2.09)		253 (2.60)
Indications, n (%)	773 (95.20)		7007 (71.96)	
Epilepsy		565 (73.09)		2311 (33.00)
Others		208 (26.91)		4696 (67.00)
Outcomes, n (%)	812 (100)		9738 (100)	
Nonserious outcome		73 (8.99)		2504 (25.71)
Serious outcome		739 (91.00)		7234 (74.29)
Death		129 (17.46)		1024 (14.16)
Life-threatening		107 (14.48)		252 (3.48)
Hospitalization		319 (43.17)		2844 (39.31)
Disability		13 (1.76)		156 (2.16)
Other-serious outcomes		546 (73.88)		4718 (65.22)
Time-to-onset (days)	218 (26.85)		1483 (15.23)	
Median (IQR)		6 (0–107.75)		12 (0–128.50)
Reporter, n (%)	807 (99.38)		9676 (99.36)	
Health professional		604 (74.85)		4799 (49.60)
Consumer		203 (25.15)		4877 (50.40)
Reporting years, n (%)	812 (100)		9738 (100)	
2022	75 (9.24)		1118 (11.48)	
2021	121 (14.90)		1850 (19.00)	
2020	79 (9.73)		801 (8.23)	
2019	107 (13.18)		1728 (17.74)	
2018	110 (13.55)		1097 (11.27)	
2017	65 (8.00)		632 (6.49)	
2016	46 (5.67)		559 (5.74)	
2015	89 (10.96)		1057 (10.85)	
2014	46 (5.67)		332 (3.41)	
2013	74 (9.11)		570 (5.85)	

*LCM* lacosamide, *AEs* adverse events, *n* number of cases, *IQR* interquartile range, *US* the United States, *JP* Japan, *DE* Germany, *FR* France, *CO* Colombia.

### Disproportionality analysis

**Figure 1:**
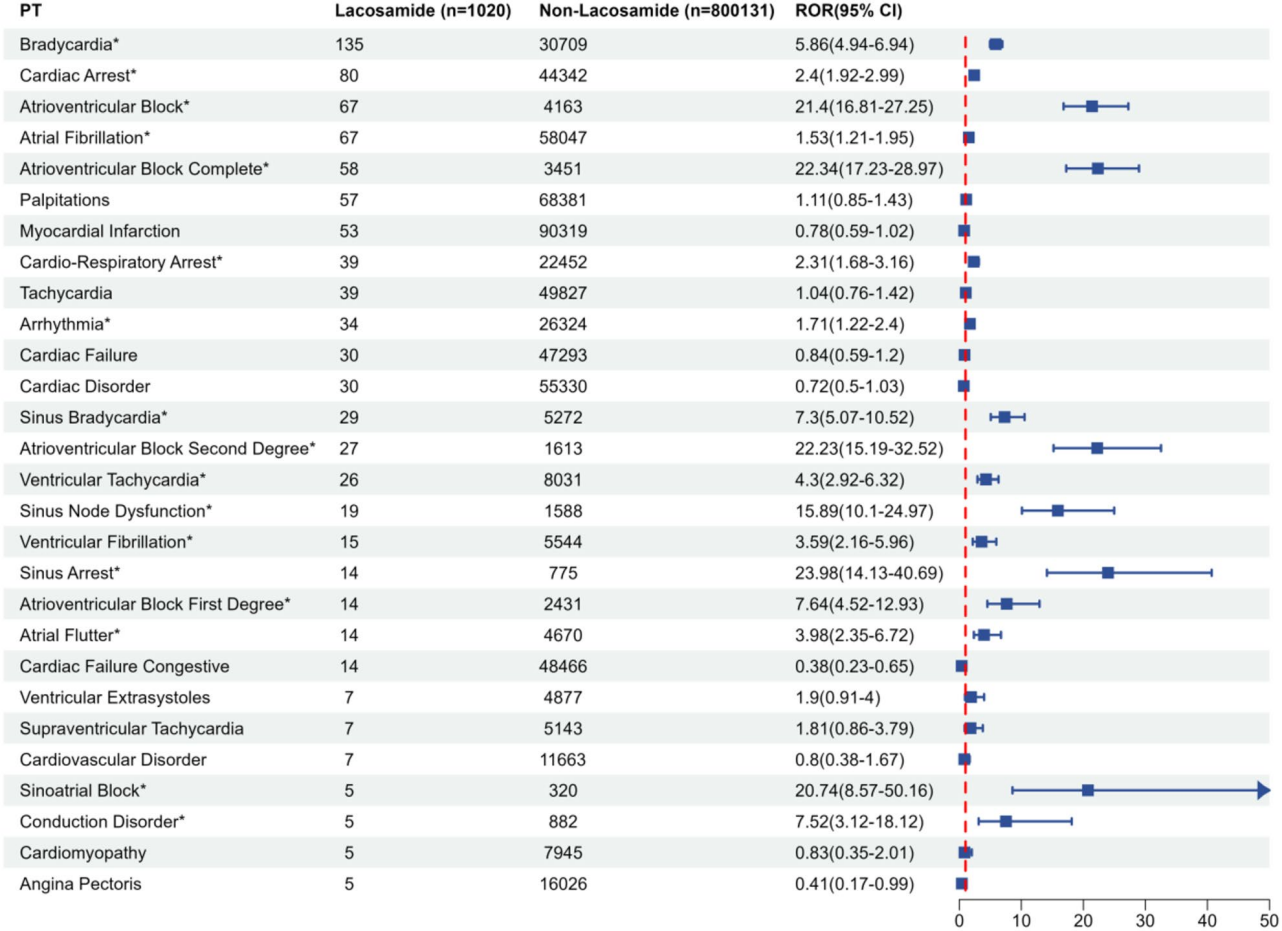
ROR with 95% CI for LCM-associated cardiac AEs with at least 5 counts. *CI* confidence interval, *n* number of cases of total AEs associated with the given drug, *ROR* reporting odds ratio; *Emerging findings of LCM- associated cardiac AEs from FAERS database.

The FAERS database from 2013 to 2022 were documented 812 cases of cardiac AEs and 92 distinct preferred terms (PTs) associated with cardiac AEs following the use of LCM. Among these, 28 PTs were noted to have reported at least 5 such AEs post-LCM application (Fig. 1). It is important to note that several events were reported for a single case or patient, meaning these figures might represent multiple counts for the same individual. Furthermore, this analysis included 812 cases of cardiac AEs, involving a total of 1020 PTs. The most commonly reported cardiac AEs were bradycardia (n = 135), cardiac arrest (n = 80), atrioventricular block (n = 67), and atrial fibrillation (n = 67). Additionally, there were 17 cardiac AEs with statistically significant signal intensity the lower limit of the reporting odds ratios (ROR) 95% CI (ROR_025_) exceeded 1. Atrial fibrillation (ROR_025_ = 1.21) is the lowest and complete atrioventricular block (ROR_025_ = 17.23) is the highest. Furthermore, Table S1 presents the non-significant signals related to cardiac AEs associated with LCM, indicated by a ROR_025_ of less than 1 or fewer than 5 instances of LCM-related cardiac AEs. The signal intensity results of LCM on System Organ Class (SOC) shown in Table S2 and the ROR with 95% CI of cardiac disorders was 1.57 (1.48–1.68).

### Serious vs. nonserious cases

The data in Table 2 indicated that the differences in gender (χ^2^ = 0.547, *p* = 0.459) and body weight (68 versus 65 kg; *p* = 0.812) between serious and non-serious cardiac AE cases were not statistically significant after the application of LCM. The exception was age (63 versus 43.5 years; P = 0.01), which was associated with more severe cardiac AE cases. Among the reported cardiac AEs, Bradycardia (χ^2^ = 9.30, *p* = 0.002) and Cardiac Arrest (χ^2^ = 5.6, *p* = 0.018) were significantly more often reported as serious events. Conversely, fifteen other AEs demonstrated no significant difference in their classification as serious or non-serious, with p-values greater than 0.05. This group includes conditions such as myocardial infarction (*p* = 0.079), atrial fibrillation (χ^2^ = 2.66, *p* = 0.103), and atrioventricular block (χ^2^ = 2.02, *p* = 0.156), among others. Notably, all AEs outcomes for atrioventricular block complete (n = 58), cardio-respiratory arrest (n = 39), palpitations (n = 38), tachycardia (n = 34), arrhythmia (n = 34), cardiac failure (n = 30), cardiac disorder (n = 29), ventricular tachycardia (n = 26), atrioventricular block second degree (n = 26), and sinus node dysfunction (n = 19) were reported as not statistically significant between serious cases and non-serious cases.

**Table 2:** Differences in clinical characteristics of serious and nonserious reports.

	Serious cases(n = 739)	Nonserious cases(n = 73)	Statistics	*P* value
Gender, n (%)			0.547^c^	0.459^a^
Female	358 (48.44)	30 (41.10)		
Male	313 (42.35)	33 (45.21)		
Age, years (Median)	63 (56.50)	43.5 (51.63)	6.05^b^	0.01^e^
Weight, Kg (Median)	68 (68.50)	65 (67.54)	0.057^b^	0.812^e^
Types of AEs, n (%)				
Bradycardia	111	1	9.30^c^	0.002^a^
Cardiac arrest	80	1	5.60^c^	0.018^a^
Atrial fibrillation	67	2	2.66^c^	0.103^a^
Atrioventricular block	60	2	2.02^c^	0.156^a^
Atrioventricular block complete	58	2	1.84^c^	0.175^a^
Myocardial infarction	53	1	–	0.079^d^
Cardio-respiratory arrest	39	1	–	0.249^d^
Palpitations	38	1	–	0.245^d^
Tachycardia	34	1	–	0.358^d^
Arrhythmia	34	2	–	0.763^d^
Cardiac failure	30	1	–	0.35^d^
Cardiac disorder	29	1	–	0.509^d^
Ventricular tachycardia	26	1	–	0.502^d^
Atrioventricular block second degree	26	2	–	1^d^
Sinus node dysfunction	19	1	–	1^d^
Sinus bradycardia	16	1	–	1^d^
Ventricular fibrillation	15	1	–	1^d^
Cardiac failure congestive	14	1	–	1^d^
Atrial flutter	14	2	–	0.648^d^
Atrioventricular block first degree	14	2	–	0.648^d^
Sinus arrest	14	1	–	1^d^
Cardiovascular disorder	7	1	–	0.531^d^
Supraventricular tachycardia	7	1	–	0.531^d^
Ventricular extrasystoles	7	1	–	0.531^d^
Angina pectoris	5	2	–	0.125^d^
Cardiomyopathy	5	1	–	0.433^d^
Sinoatrial block	5	1	–	0.433^d^
Sinus Tachycardia	4	1	–	0.376^d^
Myocardial ischaemia	4	1	–	0.376^d^
Pulseless electrical activity	4	1	–	0.376^d^
Bundle branch block left	4	1	–	0.376^d^

The AEs, listed above were AEs, with significant signal strengths. p ˂ 0.05 were considered statistically significant.

*AEs* adverse events, *n* number of cases.

^a^Proportions were compared using the Pearson χ^2^ test.

^b^The t-statistic of the independent samples t test.

^c^The χ^2^ statistic of the Pearson chi-square test.

^d^Fisher’s exact test.

^e^Mann–Whitney U test.

### Subgroup analysis

**Figure 2:**
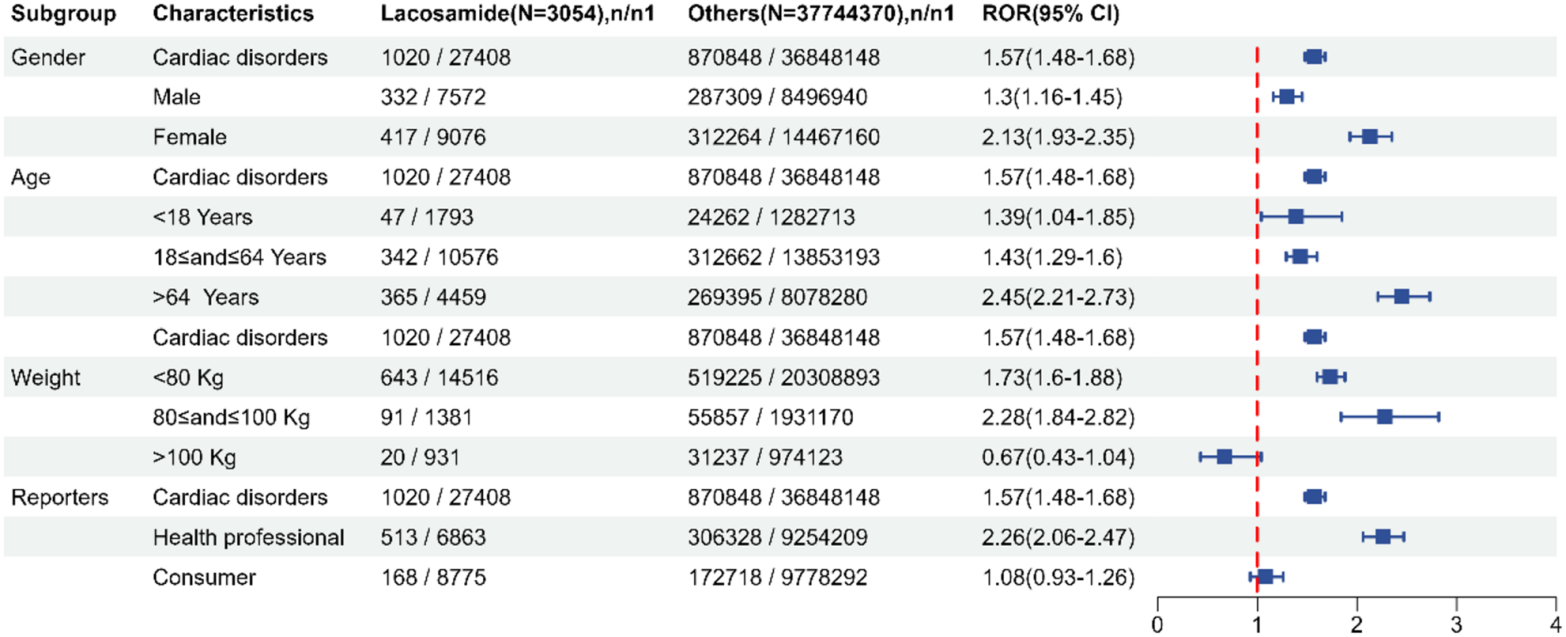
The stratification analysis of LCM-related cardiac disorders. *CI* confidence interval, *n* number of cases of total AEs associated with the given drug, *ROR* reporting odds ratio, *nl*, number of cases without suspected AEs (i.e., total AEs excluding suspected ones) associated with the given drug.

As depicted in Fig. 2, cardiac disease was sub-grouped by age, weight, sex, and reporter type, respectively. It indicates that different subgroups (such as sex, age, weight, and reporter type) have a strong statistical association with the occurrence of LCM-related cardiac disease. In gender stratification, ROR was 1.30 for males and 2.13 for females, suggesting a significant difference both in males and females. From the perspective of the age group, the ROR value of the age group over 64 years old was the largest, which was 2.45, indicating that patients in this age group were more prone to cardiac AEs after receiving LCM therapy. In the weight stratification, there were significant differences among different body weights, and the ROR value in the 80–100 kg group was 2.28. After grouping by reporter type, it could be seen that there is a more significant difference ROR 2.26 in the reports of professionals, while consumers show ROR 1.08. This suggested that differences in LCM-related cardiac AEs primarily manifested in professionally reported AEs. Table S3 records the disproportionation results of cardiac-related PTs grouped by sex, age, weight and reporter type. The results indicated that cardiac disorders exhibited variations in their stratification.

### Clinical prioritization of the disproportionality signals

Out of the 17 AEs with statistically significant disproportionality signals, a total of 15 AEs (88.24%) were categorized as important medical events (IMEs), while only ventricular fibrillation represented designated medical events (DMEs) at a rate of 5.88% (Table 3). Based on the clinical priority assessment, 4 AEs (23.53%) were identified as having weak clinical priority (0–4 scores), 13 AEs (76.47%) as having moderate clinical priority (5–7 scores), and no cardiac AEs were classified as having strong clinical priority. Among the moderate clinical priority AEs, atrioventricular block (n = 67, ROR_025_ = 16.80, and priority score = 7), atrioventricular block complete (n = 58, ROR_025_ = 17.23, and priority score = 7), and ventricular fibrillation (n = 15, ROR_025_ = 2.16, and priority score = 7) obtained the highest priority scores of 7. Additionally, 15 AEs were evaluated as having strong clinical evidence with a designation of "++". The type of AE with more deaths was respiratory arrest (n = 27), followed by cardiac arrest (n = 23). Notably, four AEs were identified as new and unexpected signals, and they were sinus node dysfunction, ventricular fibrillation, sinus arrest and sinoatrial block.

**Table 3:** Clinical priority assessing results of disproportionality signals.

PTs	N	ROR_025_	Death (n)	IMEs-DMEs	Relevant evidence evaluation	Priority level (score)
Bradycardia	135	4.94	4	IME	++	6
Cardiac arrest	80	1.92	23	IME	++	6
Atrioventricular block	67	16.80	8	IME	++	7
Atrial fibrillation	67	1.21	2	IME	+	4
Atrioventricular block Complete	58	17.23	5	IME	++	7
Cardio-respiratory arrest	39	1.68	27	NA	+	4
Arrhythmia	34	1.22	3	IME	++	4
sinus Bradycardia	29	5.07	0	IME	++	6
Atrioventricular block second degree	27	15.19	1	IME	++	5
Ventricular tachycardia	26	2.92	2	IME	++	5
Sinus node dysfunction^a^	19	10.10	1	IME	++	6
Ventricular Fibrillation ^a^	15	2.16	4	DME	++	7
Sinus Arrest ^a^	14	14.13	0	IME	++	6
Atrioventricular block first degree	14	4.52	3	IME	++	5
Atrial flutter	14	2.35	0	IME	++	5
Sinoatrial block^a^	5	8.57	0	IME	++	5
Conduction disorder	5	3.12	0	IME	++	4

*NA* not applicable (for relevant criteria), *n* number of cases, *PTs* preferred Terms, *ROR*_*025*_ the lower limit of 95% confidence interval of ROR. A priority score between 0–4, 5–7 or 8–10 represents the weak, moderate or strong clinical priority signals, respectively.

^a^New and unexpected signals, not previously reported in the drug label, emerging findings from FAERS database.

### Time-to-Onset (TTO) Analysis

As shown in Table 4, among the 17 AEs with statistically significant disproportionation signals, 13 AEs and 4 AEs were rated as moderate and weak clinical priority, respectively. The results shown that the median TTO of moderate signal AEs associated with LCM was 10 (IQR 1–153) days, while the median TTO of weak signal AEs was longer at 54 days (IQR 3–441.5). Both the shape parameter β and the upper limit of its 95% confidence interval were less than 1 in the Weibull shape parameter (WSP) test, indicating that both moderate and weak clinical priority signals demonstrate an early failure mode and a gradual decline in the likelihood of cardiac AEs over time.

**Table 4:** Results of TTO analysis for signals with moderate/weak prioritization.

Prioritization	AEs	Cases	TTO (days)		Weibull distribution				
					Scale parameter		Shape parameter		Failure type
	n1	n2	Median (IQR)	Min–max	α	95%CI	β	95%CI	
Moderate	13	119	10 (1–153)	0–3226	102.77	56.53–149.01	0.48	0.40–0.55	Early failure
Weak	4	31	54 (3–441.5)	0–1778	173.92	40.34–307.51	0.52	0.37–0.67	Early failure

*n1* number of PTs, *n2* number of cases with available time-to-onset, *IQR* interquartile range, *TTO* time-to-onset. When TTO, is 0 days, the adverse events occurred within the same day with the therapy.

## Discussion

This disproportionality study aimed to evaluate the association between LCM and cardiac AEs through data mining of the FAERS Database. Chinnasami et al. have reported a case of sinus node dysfunction observed in a 49-year-old woman with drug-resistant epilepsy after taking LCM, which resolved upon discontinuation but reappeared upon reintroduction of the medication at a lower dose^18^. Experimental studies and clinical trials indicate that LCM affects neuronal and cardiac activity, potentially raising the likelihood of cardiac arrhythmias^19^. Like other anticonvulsant drugs, the dose-dependent inhibition of sodium channels by LCM may lead to delays in cardiac conduction below the level of the His bundle, potentially contributing to the development of cardiac arrhythmias^8,11^. The available studies on cardiac AEs associated with LCM use have limitations in sample size and lack of data stratification by sex, age, and weight, highlighting the importance of ongoing post-marketing surveillance. The results provide valuable insights into the characteristics, disproportionality analysis, TTO, serious outcomes, subgroup analysis, and clinical prioritization of LCM-induced cardiac AEs.

A descriptive analysis of LCM-related cardiac AEs in the FAERS database from 2013 to 2022 identified 92 distinct PTs related to cardiac AEs (Table S1), of which 28 PTs had at least 5 reported cases. The most commonly reported cardiac AEs were bradycardia, cardiac arrest, atrioventricular block, and atrial fibrillation. Signal strength analysis indicated significant disproportional reporting of 17 cardiac AEs in association with LCM treatment. While the majority of cardiac AEs identified in this study are known to be associated with LCM, the observed frequency of these events in FAERS data was unexpectedly high. The cause might be attributed to the pharmacological action of LCM, or it could be a result of the exacerbation of the patient's pre-existing heart conditions. This finding prompts further investigation into the nature of these AEs and suggests a potential need for heightened awareness and careful monitoring.

In the study, it was observed that LCM-induced cardiac AEs were more commonly reported in females compared to males, with females accounting for 52.86% of all reports. This is consistent with previous reports^10^. Regarding age distribution, while the data indicate that patients aged 18–64 years represent 47.31% of the reported cases of cardiac AEs, a more comprehensive analysis reveals an interesting pattern. The occurrence of serious outcomes, including deaths, in a significant number of cases is a matter of concern. In addition, deaths were also reported in a multicenter long-term observational clinical study of LCM^10^, so the observation of serious adverse reactions caused by LCM should be further strengthened. Kim et al. reviewed medical records of consecutive adult epilepsy patients who received a single loading dose (400 mg) of IV LCM, 32.9% (28/85 patients) had experienced at least one cardiac AE^11^. Our mining data showed that cardiac adverse reactions had a higher proportion of serious outcomes (91% vs 74.29%) and a higher risk of life-threatening (14.48% vs 3.48%) than other adverse reactions, which is inconsistent with other studies^8,10,20^. Additionally, the median age of 49 years indicates that cardiac AEs can occur across a wide range of ages, emphasizing the need for vigilance regardless of the patient's age. The timing of onset for cardiac AEs is another crucial aspect revealed by the analysis. Approximately 8.33% of patients reported the onset time for cardiac AEs, with a median TTO of 6 days. This information suggests that vigilance should be maintained during the initial period of LCM treatment. Regarding the reporting of LCM-associated cardiac AEs, the majority of reports were submitted by healthcare professionals (74.85%), indicating their active role in identifying and reporting these events.

The analysis comparing serious and nonserious cases of cardiac AEs in LCM-treated patients revealed several notable findings. There were no significant differences in sex and body weight between the two groups, except for age, with older patients experiencing more severe cases of cardiac AEs. Bradycardia and cardiac arrest were more likely to be reported as serious AEs, in contrast other AEs, such as myocardial infarction, atrial fibrillation, and atrioventricular block, tended to be reported as nonserious AEs. These findings have implications for prevention and management strategies. Some of the findings usually associated with aging actually reflect disease processes that are more common in the elderly, heart disease being one of them, and the risk of sudden cardiac death in the elderly population is also higher^21^. In particular, elderly women are more likely to have serious cardiac adverse reactions, as Uwe Runge et al. showed^20^. AEs related to cardiac therapy in patients aged 65 years were not considered to be related to LCM. It was possible that the elderly were more likely to have diseases with cardiac symptoms.

However, other studies have shown that LCM use in the elderly does lead to serious adverse cardiac consequences, especially when given at loading dose^8,22^. In addition, serious AEs are still possible when low doses are given^23^. Healthcare professionals should be particularly vigilant in identifying and managing bradycardia and cardiac arrest cases, as these have a higher likelihood of being serious AEs. Preventive measures should focus on early detection and intervention for bradycardia and cardiac arrest. LCM is contraindicated in patients with pre-existing atrioventricular block second degree and atrioventricular block complete. It predisposes to atrial arrhythmias (atrial fibrillation or flutter), especially in patients with diabetic neuropathy and/or cardiovascular disease^16^. Electrocardiogram monitoring and evaluation of cardiac function could be beneficial for high-risk patients. Additionally, patient education regarding the symptoms of these serious AEs is crucial to promote prompt reporting and timely medical intervention.

In this study, we used a rating scale to further analyze the disproportionality signals to prioritize safety signals and avoid unnecessary warnings. The clinical prioritization analysis of LCM-associated AEs revealed that the majority of AEs were IMEs, indicating their potential impact on patient health. Ventricular fibrillation was the only disease-related DME identified. Among the AEs, 76.47% were categorized as moderate priority, with atrioventricular block (n = 67, score 7), atrioventricular block complete (n = 58, score 7), and ventricular fibrillation (n = 15, score 7) standing out as the highest priority AEs. In addition, the most frequently reported LCM-associated priority cardiac AEs were bradycardia (n = 135, score 6), cardiac arrest (n = 80, score 6), sinus bradycardia (n = 29, score 6), atrioventricular block second degree (n = 27, score 5), ventricular tachycardia (n = 26, score 5), sinus node dysfunction (n = 19, score 6), sinus arrest (n = 14, score 6), atrioventricular block first degree (n = 14, score 5), atrial flutter (n = 14, score 5), sinoatrial block (n = 5, score 5), which were consistent with the drug label. D Kropeit et al. reported that there are no findings of second degree or higher atrioventricular block following short-term adjunctive LCM therapy^19^. Case reports of cardiac adverse reactions caused by LCM were mainly in the elderly and with high doses^24,25^.

The reporting of LCM-related cardiac AEs tended to be severe AEs with strong clinical priority, which piqued our curiosity to delve deeper into its specific characteristics. A total of 111, 80, 67, 60 and 58 cases of LCM–associated bradycardia, cardiac arrest, atrial fibrillation, atrioventricular block, and atrioventricular block complete were collected from the FAERS database, with 1, 1, 2, 2, and 2 reported as serious cases, respectively (Table 2). Consistent with one case report that descripted the case of an old female patient with significant cardiac and renal risk factors had a reversible and complete block of AV conduction after receiving a high dose of intravenous LCM^13^. Therefore, clinicians should promptly alert patients to watch for symptoms after LCM administration to mitigate risks, particularly for those with pre-existing cardiac conditions who require appropriate monitoring. All disproportionality clinical priority signals of LCM treatment were compiled in Table 3. The use of Weibull parameters to predict AE occurrence provided valuable insights for patient pharmacological management in clinical practice^26^. In the TTO analysis, the median TTO for moderate-priority AEs was 10 days, with an IQR of 1 to 153 days. Likewise, the median time to onset of weak-priority AEs was 54 days, ranging from 3 to 441.5 days according to IQR. The results of the WSP test showed that both the shape parameter β and the upper limit of its 95% confidence interval were less than 1. This suggests a moderate to weak clinical priority signal exhibiting an early failure pattern, indicating that the majority of cardiac AEs associated with LCM occurred within a relatively short time after initiation of treatment. Furthermore, the risk of cardiac AEs gradually decreased over time, highlighting the downward trend in the incidence of these events. This result exits some unavoidable biases due to inherent limitations such as the study lacks sufficient evidence to prove causality.

Our finding is consistent with two studies that demonstrated a dose-dependent relationship and a gradual decline in cardiac AEs associated with LCM over time^8,11^. Hence, vigilant monitoring during the initial period following LCM administration could effectively detect most cardiac AEs. If cardiac AEs are identified in patients, timely dose adjustment or implementation of appropriate supportive measures can help alleviate symptoms and prevent the occurrence of severe AEs. Notably, out of the 17 cardiac AEs, four AEs were identified as new and unexpected signals that were not previously reported on the drug label^16^. These new signals included sinus node dysfunction, ventricular fibrillation, sinus arrest and sinoatrial block. To confirm the newly identified cardiac AEs associated with LCM treatment, such as arrhythmia and ventricular fibrillation, it is essential to conduct detailed data analysis of existing patient records and enhance post-marketing surveillance to monitor these events in real-time. Collaboration with healthcare professionals for improved reporting of AEs and incorporating patient-centric research approaches are also vital steps in validating these signals. The specific effects of LCM on these AEs and the underlying mechanisms of their potential association have yet to be thoroughly investigated, warranting further clinical exploration. Given the widespread presence of cardiac AEs, particularly the newly recorded signals, clinicians should be alerted to these safety concerns and consider them important safety alerts.

### Limitations

All data mining efforts using the FAERS database encounter some unavoidable biases due to inherent limitations: (1) The possibility of false, inaccurate, incomplete, or delayed reporting in voluntarily submitted reports, which are difficult to rectify; (2) The lack of information on the total population using LCM, preventing the calculation of AE incidence rates; (3) This study employed disproportionality analysis to provide evidence of associations between the target drug and AEs, lacking sufficient evidence to prove causality. Moreover, our research focused solely on cardiac-related AEs, leaving the deeper connections with AEs in other systemic organs unclear. Further clinical trial studies are needed to validate the findings.

## Conclusions

Our disproportionality study conducted a comprehensive and systematic investigation into the potential cardiotoxicity of LCM, providing novel safety insights. Among the 17 cardiac AEs significantly associated with LCM treatment, we confirmed the presence of 4 new and unexpected signals. Additionally, we identified 13 cardiac AEs with moderate clinical priority and 4 with weak clinical priority. The median TTO for moderate clinical priority signals was 10 days, and 54 days for weak clinical priority signals. Notably, all cardiac AEs exhibited characteristics of early failure types. Older patients experienced more severe cases of cardiac AEs, and bradycardia and cardiac arrest were more frequently reported as serious AEs. The findings of this study shed light on the spectrum of cardiotoxicity associated with LCM, enabling clinicians to distinguish different manifestations of cardiotoxicity. Some cardiac AEs have not received sufficient attention, emphasizing the need for increased cardiac examinations, especially in high-risk patients. By staying vigilant and proactive in monitoring patients receiving LCM, healthcare professionals can better manage better manage LCM-related cardiac AEs.

## Methods

### Data source

LCM was selected as the study drug. The data for this analysis were obtained from the FAERS Quarterly Data Extract Files, accessed at http://www.fda.gov/. The FAERS database is publicly available to all users. To ensure the inclusion of the most recent reports, we extracted all relevant data recorded in FAERS database from 2013 Q1 to 2022 Q4 for our study.

### Data extraction

**Figure 3:**
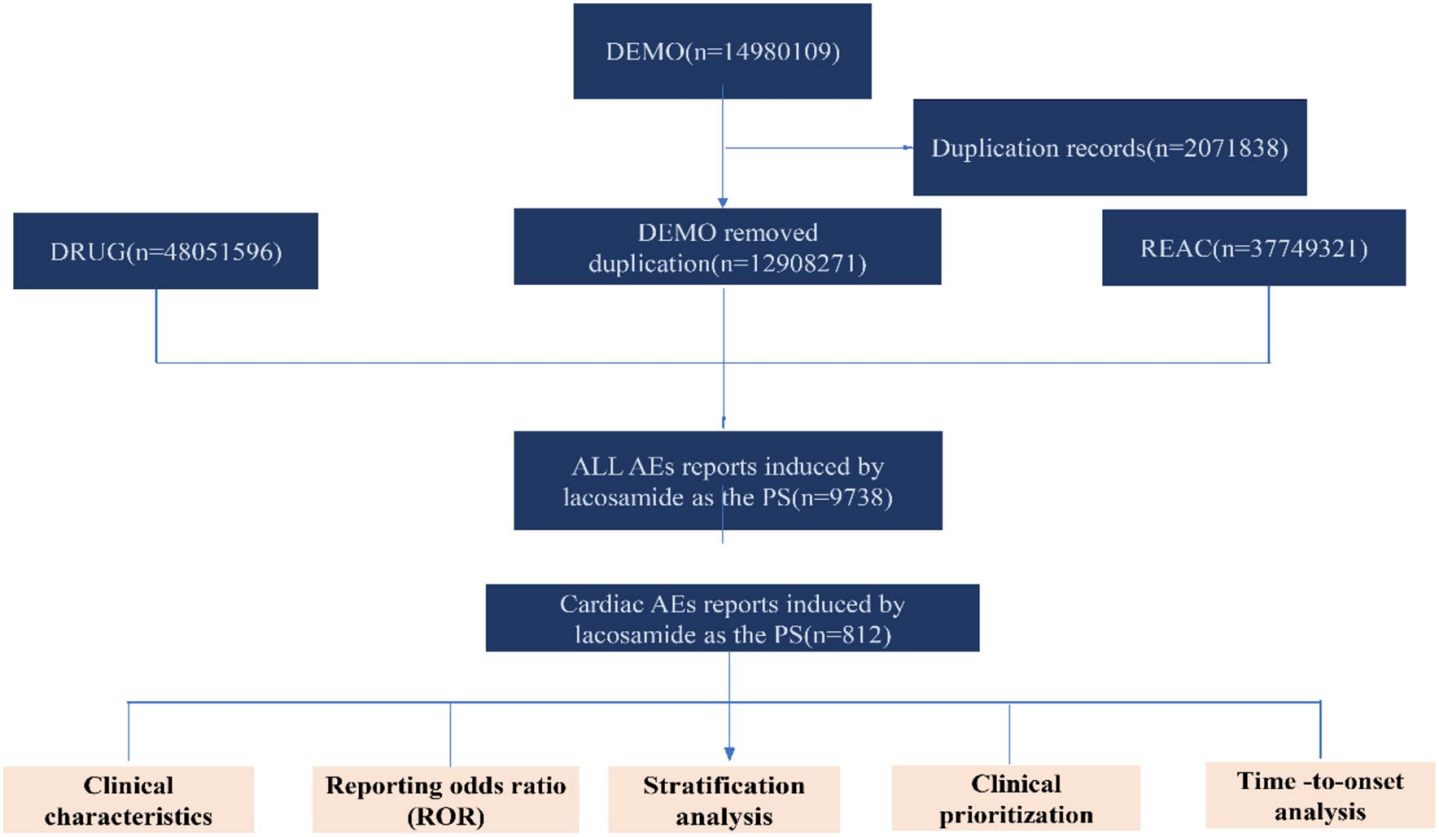
The process of selecting LCM-associated cardiac AEs from FAERS database.

A disproportionality analysis was employed to quantify the association between LCM and cardiological AEs. This analysis measures the occurrence of the specific AEs related to LCM compared to all other drugs present in the database^27^. The study utilized 7 data files containing patient demographic information (DEMO), adverse events (REAC), drug/biologic information (DRUG), report sources (RPSR), patient outcomes (OUTC), indications for drug (INDI) and start/end dates of drug therapy (THER) to conduct a comprehensive analysis^28^. A relationship was established to link each data file using specific identification numbers (e.g., caseid, primaryid) in the FAERS database architecture, and duplicate reports were eliminated before conducting the statistical analysis to ensure report uniqueness^29^. Following FDA guidelines, duplicate records were removed using key filters, namely caseid, fda_dt, and primaryid. The caseid with the largest fda_dt was selected first, and in cases where the caseid and fda_dt were the same, the report with the higher primaryid was chosen. Reports were extracted using the generic name (LACOSAMIDE in drugname and prod_ai columns) and trade name (VIMPAT in drugname column) from the DRUG file. To enhance the association between the drug and AEs, only role_cod as primary suspected (PS) was considered. AEs coded using the PT from the standardized Medical Dictionary for Regulatory Activities (MedDRA 26.0) were analyzed, excluding PTs related to off-label use, product issues, medication and other product use errors, and epilepsy disorders. Reports with input errors (EVENT_DT earlier than START_DT) were excluded prior to the analysis to ensure the accuracy of the calculations. The analysis focused on calculating all PTs falling under the SOC of cardiac disorders (SOC: 10007541) in MedDRA 26.0. All data processing and analyses were performed using SPSS (v22.0; IBM Corp., Armonk, NY, United States) and R software (version 4.0.5; https://www.r-project.org/). The data extracted this time was extracted with LCM as the main suspected drug, and the extracted records of AEs reported with LCM as the main suspected drug were 9738 cases. This flowchart in Fig. 3 depicted the multi-step process of data extraction, processing, and analysis, if several events may be reported for a single case, the case will be double-counted in the analysis of data.

The study provided a comprehensive description of the clinical characteristics of the reports, encompassing details such as gender, age, weight, reporting area, indications, outcomes, and reporters. Notably, serious outcomes were categorized as death, life-threatening situations, hospitalization, disability, and other critical conditions. Means (± standard deviation) were employed to represent continuous variables with a normal distribution, while proportions were used for categorical variables.

### Statistical analysis

All cardiological AE reports within the FAERS database were selected for signal intensity analysis of LCM reports at the PT level (cardiological AEs) and the SOC level (cardiac disorders). The ROR was determined through a disproportionality analysis, utilizing a 2 × 2 table (Table 5), to identify any potential association between a drug and an AE^30^. To ensure reliable signals, we defined significant associations when the lower limit of the ROR 95% CI exceeded 1, and at least 5 reports were present. AE outcomes reported in FAERS were categorized as serious and nonserious to assess the severity of detected safety signals and identify potential risk factors (gender, age, and weight) in patients. Seriousness outcomes included death (DE), life-threatening situations (LT), congenital anomalies (CA), disability (DS), inpatient hospitalization or prolongation of existing hospitalization (HO), required intervention to prevent permanent impairment/damage (RI), as well as other serious/important medical events (OT). Some reports may list multiple specific outcomes (e.g., DS, LT, and DE), which were recorded in the OUTC file. Furthermore, we compared serious and nonserious reports to better understand the severity of detected safety signals and identify potential risk factors (gender, age, weight, and types of AEs) in patients. Proportions were compared using Pearson's chi-squared (χ^2^) or Fisher's exact test, while an independent samples t-test was utilized for continuous data such as age and weight. The data were analyzed using SPSS, with statistical significance set at a two-tailed *p* < 0.05. To investigate the impact of different stratification schemes on the correlation between LCM and cardiac disorders, we conducted subgroup analyses based on sex (female and male), age categories (< 18, 18 ≤ and ≤ 64, > 64 years), weight categories (< 80, 80 ≤ and ≤ 100, and > 100 kg), and reporter type (health-care professionals and consumers). These subgroup analyses aimed to further explore the associations between LCM and cardiac disorders within specific patient subgroups.

**Table 5:** The 2 × 2 contingency table.

	PTs of interest	All other PTs	Total
Drug of interest	a	b	a + b
All other drugs in FAERs	c	d	c + d
Total	a + c	b + d	N = a + b + c + d

a: the number of PTs caused by suspected AE for the suspect drug; b: the number of PTs caused by all other AEs for the suspect drug; c: the number of PTs caused by suspected AEs for all other drugs; d: the number of PTs caused by all other AEs for all other drugs.

*N* total number of AE reports in the FAERS database, *AEs* adverse events, *FAERS* FDA adverse event reporting system.

In our study, AEs with significant disproportionality were categorized into three types based on clinical priority: weak clinical priority, moderate clinical priority, and strong clinical priority. A semi-quantitative score was implemented to prioritize disproportionality signals, considering five distinct features: the number of target AEs, ROR_025_ values, mortality proportion, characterization as IMEs or DMEs, and evidence evaluation^31,32^. The categorization of IME/DME is determined according to guidelines provided by the EMA. This agency outlines specific criteria for inclusion or exclusion under the category of “IMEs” and delineates “DMEs” as encompassing medical conditions that are inherently serious and frequently associated with medications^33^. Refer to Table S4 for detailed information regarding these prioritization criteria.

### TTO analysis

TTO for a specific event was computed as the duration between the occurrence date of AEs (EVENT_DT in DEMO file) and the start date of LCM use (START_DT in THER file)^34^. Only the reports that contained TTO data were considered. The incidence of AEs often varies over time, and statistical analysis of TTO uses the WSP test to describe the risk of increasing or decreasing the incidence of AEs over time^26,35^. The Weibull distribution was characterized by two parameters: scale (α) and shape (β). The median TTO and WSP were calculated to predict the risk of an increase or decrease in AEs with weak or moderate clinical priority over time following the initiation of LCM therapy. Previous studies provided the selection criteria for these parameters and their evaluation. In the WSP test, the shape parameter β of the Weibull distribution was considered to predict the hazard in the absence of a reference population. The WSP test describes three types of hazards: an early failure type indicates that the hazard of AEs decreases over time (β < 1 and 95% CI < 1); a random failure type suggests that the hazard of the AEs remains constant over time (β is equal to or nearly 1, and its 95% CI includes the value 1); and a wear-out failure type signifies that the hazard of the AEs increases over time (β > 1 and 95% CI > 1). To accomplish this prediction for LCM-associated cardiological AEs over time, Minitab statistical software (v20.0; Minitab LLC, State College, PA, United States) was utilized to perform the WSP tests.

### Supplementary Information

Supplementary Tables.

## Data Availability

The datasets generated during and/or analyzed during the current study are available from the corresponding author upon reasonable request.
