# Supplementary material for: Cardiac adverse events associated with lacosamide: a disproportionality analysis of the FAERS database
Source: Sci Rep. 2024 Jul 13;14:16202. doi: 10.1038/s41598-024-67209-0 (PMC11246456; doi:10.1038/s41598-024-67209-0)
Supplement: Supplementary file 1 — Supplementary Tables. [file 41598_2024_67209_MOESM1_ESM.pdf]

Supporting Information for

Cardiac adverse events associated with Lacosamide: A Pharmacovigilance Study of the FAERS Database

Chengcheng Yang [a], Wanqi Zhao [b], Huihui Chen [a], Yinhui Yao [c], Jingmin Zhang [d*]

[a]*Department of Pharmacy, The First People's Hospital of Shangqiu, Shangqiu, 476000, China.*

[b]*The University of Manchester, Manchester M13 9PL, England.*

[c]*Department of Pharmacy, Chengde Medical University Affiliated Hospital, Chengde, 067000, China.*

[d]*Department of Pharmacy, Henan Key Laboratory for Precision Clinical Pharmacy, The First Affiliated Hospital of Zhengzhou University, Zhengzhou, 450052, China*

* Corresponding author

Jingmin Zhang

Department of Pharmacy, Henan Key Laboratory for Precision Clinical Pharmacy, The First Affiliated Hospital of Zhengzhou University, Zhengzhou, 450052, China;

E-mail: fcczhangjm@zzu.edu.cn

TABLES

Table S1 Other lacosamide-related nonpositive cardiac AE signals

PT	Lacosamide-related cardiac AE (n)	ROR (95%*CI*)
Palpitations	57	1.11 (0.85-1.43)
Myocardial Infarction	53	0.78 (0.59-1.02)
Tachycardia	39	1.04 (0.76-1.42)
Cardiac Disorder	30	0.72 (0.50-1.03)
Cardiac Failure	30	0.84 (0.59-1.20)
Cardiac Failure Congestive	14	0.38 (0.23-0.65)
Cardiovascular Disorder	7	0.80 (0.38-1.67)
Supraventricular Tachycardia	7	1.81 (0.86-3.79)
Ventricular Extrasystoles	7	1.90 (0.91-4.00)

Angina Pectoris	5	0.41 (0.17-0.99)
Cardiomyopathy	5	0.83 (0.35-2.01)
Sinus Tachycardia	4	0.73 (0.27-1.93)
Myocardial Ischaemia	4	0.92 (0.35-2.46)
Pulseless Electrical Activity	4	1.88 (0.71-5.02)
Bundle Branch Block Left	4	2.44 (0.92-6.51)
Acute Myocardial Infarction	3	0.23 (0.08-0.72)
Pericardial Effusion	3	0.30 (0.10-0.94)
Coronary Artery Occlusion	3	0.58 (0.19-1.80)
Cardiotoxicity	3	0.76 (0.25-2.36)
Torsade De Pointes	3	0.98 (0.31-3.03)
Cardiac Failure Acute	3	1.02 (0.33-3.16)
Extrasystoles	3	1.05 (0.34-3.27)
Stress Cardiomyopathy	3	1.19 (0.38-3.68)
Arteriosclerosis Coronary Artery	3	1.26 (0.41-3.91)
Bundle Branch Block Right	3	1.70 (0.55-5.29)
Ventricular Arrhythmia	3	1.84 (0.59-5.70)
Aortic Valve Incompetence	3	2.10 (0.68-6.51)
Bradyarrhythmia	3	4.63 (1.49-14.38)
Nodal Rhythm	3	5.79 (1.86-18.01)
Sinus Arrhythmia	3	6.09 (1.96-18.92)
Pericarditis	2	0.31 (0.08-1.26)
Cardiogenic Shock	2	0.35 (0.09-1.41)
Myocarditis	2	0.44 (0.11-1.76)
Cardiomegaly	2	0.44 (0.11-1.78)
Cardiac Flutter	2	0.69 (0.17-2.77)
Tricuspid Valve Incompetence	2	0.79 (0.20-3.14)
Cardiopulmonary Failure	2	1.23 (0.31-4.91)
Supraventricular Extrasystoles	2	1.69 (0.42-6.76)
Arrhythmia Supraventricular	2	5.10 (1.27-20.46)
Nodal Arrhythmia	2	6.79 (1.69-27.24)
Adams-Stokes Syndrome	2	34.02 (8.36-138.47)
Trifascicular Block	2	39.03 (9.56-159.25)
Coronary Artery Disease	1	0.09 (0.01-0.61)
Mitral Valve Incompetence	1	0.26 (0.04-1.87)
Left Ventricular Dysfunction	1	0.34 (0.05-2.39)
Cardiac Dysfunction	1	0.64 (0.09-4.51)
Intracardiac Thrombus	1	0.71 (0.10-5.01)
Diastolic Dysfunction	1	0.79 (0.11-5.64)
Ventricular Hypokinesia	1	0.81 (0.11-5.78)
Ischaemic Cardiomyopathy	1	0.84 (0.12-5.98)
Tachyarrhythmia	1	0.96 (0.14-6.82)
Cardiac Discomfort	1	1.00 (0.14-7.13)
Myocardial Injury	1	1.14 (0.16-8.13)

Aortic Valve Stenosis	1	1.25 (0.18-8.86)
Atrial Tachycardia	1	1.40 (0.20-9.93)
Mitral Valve Disease	1	1.41 (0.20-10.03)
Cardiac Fibrillation	1	1.64 (0.23-11.67)
Mitral Valve Prolapse	1	1.72 (0.24-12.20)
Systolic Dysfunction	1	1.77 (0.25-12.59)
Bradycardia Neonatal	1	1.81 (0.25-12.85)
Right Ventricular Hypertrophy	1	2.19 (0.31-15.60)
Postural Orthostatic Tachycardia Syndrome	1	2.26 (0.32-16.07)
Foetal Heart Rate Deceleration Abnormality	1	2.38 (0.33-16.94)
Bundle Branch Block	1	2.91 (0.41-20.70)
Right Ventricular Dilatation	1	3.16 (0.44-22.48)
Cardiovascular Symptom	1	4.81 (0.67-34.25)
Chronic Left Ventricular Failure	1	5.69 (0.80-40.60)
Atrial Enlargement	1	7.13 (1.00-50.91)
Myocardial Depression	1	8.29 (1.16-59.24)
Right Atrial Enlargement	1	8.79 (1.23-62.79)
Cardiac Sarcoidosis	1	13.54 (1.89-97.09)
Ventricular Asystole	1	13.54 (1.89-97.09)
Neonatal Cardiac Failure	1	31.59 (4.35-229.55)
Baseline Foetal Heart Rate Variability Disorder	1	34.02 (4.67-247.65)
Rebound Tachycardia	1	73.72 (9.84-552.22)

CI, confidence interval; n, number of cases of total AEs associated with the given drug;ROR, reporting odds ratio.

Table S2 Results of signal strength of lacosamide at the SOC level

**SOC**	**Lacosamide-related SOC (n)**	**ROR (95%*CI*)**
Nervous system disorders	8040	4.48 (4.37-4.60)
Injury, poisoning and procedural complications	5038	1.78 (1.73-1.84)
General disorders and administration site conditions	3409	0.62 (0.60-0.65)

Psychiatric disorders	2398	1.60 (1.54-1.67)
Gastrointestinal disorders	1119	0.44 (0.42-0.47)
Cardiac disorders	1020	1.57 (1.48-1.68)
Infections and infestations	843	0.55 (0.51-0.59)
Investigations	842	0.49 (0.46-0.52)
Skin and subcutaneous tissue disorders	649	0.40 (0.37-0.43)
Respiratory, thoracic and mediastinal disorders	471	0.34 (0.31-0.38)
Eye disorders	471	0.85 (0.78-0.93)
Musculoskeletal and connective tissue disorders	433	0.28 (0.25-0.30)
Neoplasms benign, malignant and unspecified (incl cysts and polyps)	400	0.46 (0.42-0.51)
Metabolism and nutrition disorders	394	0.66 (0.60-0.73)
Pregnancy, puerperium and perinatal conditions	323	2.82 (2.52-3.14)
Vascular disorders	278	0.47 (0.42-0.53)
Renal and urinary disorders	210	0.37 (0.32-0.42)
Blood and lymphatic system disorders	204	0.44 (0.39-0.51)
Hepatobiliary disorders	155	0.67 (0.57-0.78)
Ear and labyrinth disorders	147	1.18 (1.01-1.39)
Immune system disorders	125	0.37 (0.31-0.45)
Reproductive system and breast disorders	79	0.32 (0.26-0.40)
Endocrine disorders	30	0.42 (0.29-0.60)

CI, confidence interval; n, number of cases of total AEs associated with the given drug;ROR, reporting odds ratio.

Table S3 Cardiotoxicity spectrums for different stratification strategies are presented as ROR_{025} (n). Blank Spaces represent not eliglible for disproportionality analysis (at least 5 reports could be included for analysis)

	Stratification strategy					
	**Female**	**Male**	**Age(18-64 years)**	**Age (≥65 years)**	**Healthcare professional**	**Consumer**
Bradycardia	4.54 (69)	4.12 (51)	3.39 (41)	10.83 (55)	10.21 (125)	0.43 (10)
Cardiac Arrest	2.14 (49)	1.43 (28)	1.83 (33)	2.84 (24)	3.24 (62)	0.62 (18)
Atrioventricular Block	12.76 (30)	13.14 (25)	8.04 (16)	24.03 (20)	32.57 (59)	1.93 (7)
Atrial Fibrillation	0.96 (31)	1.23 (31)	0.63 (17)	2.95 (31)	2.07 (53)	0.35 (14)
Atrioventricular	12.38 (25)	17.40 (27)	2.05 (5)	68.47 (41)	36.31 (55)	

Block Complete						
Cardio-Respiratory Arrest	1.47 (20)	1.65 (18)	1.75 (18)	2.12 (11)	2.77 (30)	0.51 (9)
Arrhythmia	0.95 (16)	0.85 (12)	0.71 (10)	0.80 (6)	1.72 (23)	0.56 (11)
Sinus Bradycardia	2.26 (9)	7.02 (18)	4.93 (13)	9.02 (11)	10.88 (28)	
Atrioventricular Block Second Degree	10.68 (12)	13.67 (12)	16.01 (13)	9.95 (5)	32.47 (26)	
Ventricular Tachycardia	2.63 (14)	1.90 (9)	2.93 (12)	4.56 (9)	6.54 (26)	
Sinus Node Dysfunction	7.47 (9)	11.0 (10)	7.20 (7)	26.34 (10)	22.59 (19)	
Ventricular Fibrillation	1.84 (8)	1.23 (5)	3.35 (10)		4.84 (15)	
Sinus Arrest	13.04 (8)	11.23 (6)		46.85 (9)	31.60 (14)	
Atrioventricular Block First Degree	2.18 (5)	4.47 (7)		6.67 (5)	9.20 (13)	
Atrial Flutter	1.82 (7)	1.45 (5)	1.54 (5)		5.26 (14)	
Sinoatrial Block					19.16 (5)	
Conduction Disorder					6.98 (5)	

Table S4 Semi-quantitative score assessing clinical priority of adverse events showing statistically significant disproportionality

Clinical priority features	2 points	1 point	0 points
No. of events	>50	10-50	<10
Magnitude of the lower limit of the 95% *CI* of the ROR	>5	2-5	1-2
Mortality frequency	>50%	25-50%	<25%
Important or designated medical events	Designated medical event	Important medical event	None
Biological plausibility	Established on the basis of mechanism of action of lacosamide or heart failure related	Supposed on the basis of preclinical evidence or presumed comorbidities or concomitant drugs	No clear biological plausibility can be retrieved

Table S5 Top 20 concomitant drugs for LCM- related cardiac AEs from

Concomitant drugs (TOP 20)	N（%）
Levetiracetam	243(29.93)
Valproic Acid	76(9.36)
Lamotrigine	60(7.39)
Aspirin	57(7.02)
Carbamazepine	49(6.03)
Clobazam	41(5.05)
Levothyroxine	40(4.93)
Phenytoin	39(4.80)
Clonazepam	37(4.56)
Amlodipine	36(4.43)
Furosemide	35(4.31)
Metoprolol	34(4.19)
Pantoprazole	33(4.06)
Atorvastatin	33(4.06)
Oxcarbazepine	31(3.82)
Acetaminophen	30(3.69)
Midazolam	28(3.45)
Lorazepam	27(3.33)
Topiramate	26(3.20)
Phenobarbital	25(3.08)
